# On the identification of a Pliocene time slice for data–model comparison

**DOI:** 10.1098/rsta.2012.0515

**Published:** 2013-10-28

**Authors:** Alan M. Haywood, Aisling M. Dolan, Steven J. Pickering, Harry J. Dowsett, Erin L. McClymont, Caroline L. Prescott, Ulrich Salzmann, Daniel J. Hill, Stephen J. Hunter, Daniel J. Lunt, James O. Pope, Paul J. Valdes

**Affiliations:** 1 School of Earth and Environment, University of Leeds, Woodhouse Lane, Leeds LS2 9JT, UK; 2 Eastern Geology and Paleoclimate Science Center, USGS, 926A National Center, Reston, VA 20192, USA; 3 Department of Geography, Durham University, South Road, Durham DH1 3LE, UK; 4 School of Built and Natural Environment, Northumbria University, Ellison Building, Newcastle upon Tyne NE1 8ST, UK; 5 British Geological Survey, Environmental Science Centre, Keyworth, Nottingham NG12 5GG, UK; 6 School of Geographical Sciences, University of Bristol, University Road, Bristol BS8 1SS, UK

**Keywords:** Pliocene, climate models, climate sensitivity, Earth system sensitivity

## Abstract

The characteristics of the mid-Pliocene warm period (mPWP: 3.264–3.025 Ma BP) have been examined using geological proxies and climate models. While there is agreement between models and data, details of regional climate differ. Uncertainties in prescribed forcings and in proxy data limit the utility of the interval to understand the dynamics of a warmer than present climate or evaluate models. This uncertainty comes, in part, from the reconstruction of a *time slab* rather than a *time slice*, where forcings required by climate models can be more adequately constrained. Here, we describe the rationale and approach for identifying a time slice(s) for Pliocene environmental reconstruction. A time slice centred on 3.205 Ma BP (3.204–3.207 Ma BP) has been identified as a priority for investigation. It is a warm interval characterized by a negative benthic oxygen isotope excursion (0.21–0.23‰) centred on marine isotope stage KM5c (KM5.3). It occurred during a period of orbital forcing that was very similar to present day. Climate model simulations indicate that proxy temperature estimates are unlikely to be significantly affected by orbital forcing for at least a precession cycle centred on the time slice, with the North Atlantic potentially being an important exception.

## 1. Introduction

### (a) The importance of the mid-Pliocene warm period

Compared with the Pleistocene, the mPWP represents an interval of relatively warm and stable climate between 3.264 and 3.025 Ma BP [1,2]. According to the geological time scale of Gradstein *et al.* [3], it sits within the Piacenzian Stage of the late Pliocene. The interval is synonymous with the Pliocene Research Interpretation and Synoptic Mapping (PRISM) time slab for which a global dataset of palaeoenvironmental conditions has been developed by the US Geological Survey and international collaborators [1,2]. The PRISM project has documented patterns of sea-surface temperature (SST) [4–6] and land cover [7,8] using multiple proxy techniques, as well as reconstructing deep-ocean temperatures [6]. Estimates of sea level as well as topographic differences between the mid-Pliocene and present day have been produced [9,10]. These reconstructions were developed with a dual purpose: to provide greater understanding of climate and environments in a warmer world, and to provide geographically continuous boundary conditions to facilitate Pliocene climate model experiments [1].

Until 2004, atmospheric general circulation models (AGCMs) were the only type of climate model applied in a mid-Pliocene context [11–13]. These models required global information on SST, sea-ice cover as well as land cover, as they are not predicted variables in such models. In later years, single-site SSTs and land-cover data are increasingly being used to evaluate model outputs, as climate models have developed and can now predict SSTs and vegetation (coupled atmosphere–ocean–vegetation–climate models—AOGCMs and AOVGCMs). Therefore, the use of the PRISM dataset is evolving from specifying boundary conditions in models towards a model evaluation approach [14–17].

Both geological data and model outputs have shed considerable light on the nature of mid-Pliocene climate and environments. During warm phases of the mid-Pliocene, highlighted by negative excursions in *δ*^18^O from benthic foraminifera, Antarctic and/or Greenland ice volume may have been reduced [18–22]. Between 2.7 and 3.2 Ma BP the peak sea level is estimated to have been 22±10 m higher than modern [23], and it appears that SSTs were warmer [1], particularly in the higher latitudes and upwelling zones [17,24]. Sea-ice cover also declined substantially [25–27]. On land, the global extent of arid deserts decreased and forests replaced tundra in the Northern Hemisphere [8]. Based on model predictions, the global annual mean temperature may have increased by more than 3°C [14]. Meridional and zonal temperature gradients were reduced, which had a significant impact on the Hadley and Walker circulations [13,28]. The East Asian summer monsoon as well as other monsoon systems may have been enhanced [29].

Given the abundance of proxy data, the mid-Pliocene has become a focus for data–model comparisons that attempt to analyse the ability of climate models to reproduce a warm climate state in the Earth's history [14,16,17,30]. Furthermore, the mPWP has been proposed as an important interval to assess the sensitivity of climate to current or near future concentrations of carbon dioxide (CO_2_) in the longer term (hundreds to thousands of years) [15]. This links directly to the emerging paradigm of Earth system sensitivity [15,31]. Unlike traditional climate sensitivity, which is defined by the equilibrium global mean temperature response to a doubling of atmospheric CO_2_ from short-term feedbacks (Charney sensitivity) [32], Earth system sensitivity includes feedbacks from slower responding components of the climate system, including the ice sheets and vegetation [15]. These feedbacks may eventually alter the global mean temperature response to a given change in CO_2_ concentration. Estimates of Earth system sensitivity, based on examining a past warm interval such as the Pliocene, could provide a means to develop CO_2_ emission reduction targets and climate stabilization scenarios, which would enable the global mean temperature change to remain below the European Union defined threshold of 2°C in the long term [33,34].

### (b) Limitations of a time slab approach

PRISM appreciated the challenges of providing AGCMs with a truly global dataset of environmental boundary conditions. Inherent limitations that existed at the time of correlating one marine or land site to another over vast geographical distances ruled out the identification of a discrete time slice in the Pliocene [35]. Instead, PRISM took a pragmatic approach of establishing a time slab to which the ages of marine or terrestrial sites could be more confidently attributed [35]. It also naturally increased the potential amount of geological data that could be incorporated, and would therefore underpin the environmental reconstruction.

While this approach solved one problem, it created another. Climate and environmental variation (including sea level) during the mid-Pliocene is likely to have been smaller than for the past 2 million years, yet clear variations do occur over orbital time scales [36–38]. However, in terms of boundary conditions for climate models, or for proxy temperature estimates used for climate model evaluation, a single SST value and a single land classification is generally required.

**Figure 1.**
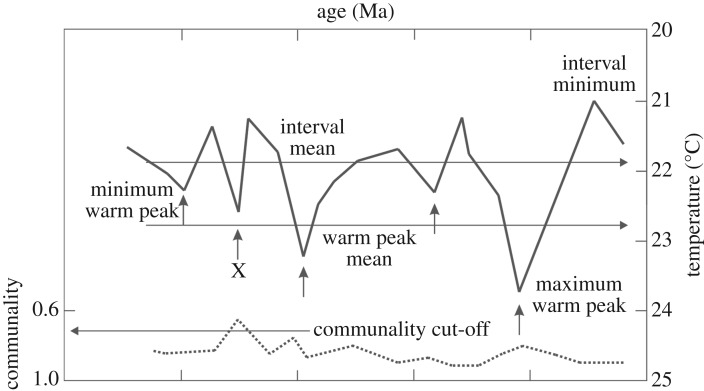
Schematic of the PRISM methodology of warm peak averaging adapted from Dowsett & Poore [35]. Idealized down-core variation in sea-surface temperature (SST) shown. Warm peak mean, warm peak minimum and warm peak maximum SST values are labelled along with minimum and mean SSTs during the interval. Communality cut-off highlighted, with peaks having a communality value of less than 0.7 being discarded (indicated by the X).

In response to this, PRISM established the methodology of SST warm peak averaging (figure 1) [35], where warm inflections in down-core measurements of SSTs are calculated. Foraminifera assemblages that achieve a sufficiently high communality cut-off (0.7 or greater) are retained and then averaged to produce a single SST value per core site [35]. On land, evidence for variability in vegetation type over orbital time scales is less common, and the window of time that has to be used to generate a satisfactory distribution of land-cover data is larger (1 million years—the entire Piacenzian Stage). If information on vegetation variability is available, then the biome representing the warmest climatic conditions has been selected and placed into the land-cover reconstruction [8].

So what exactly does the PRISM environmental reconstruction represent? From site to site, it is an average of warm climate signals that occurred during a time slab. It should not be considered as a reconstruction of environmental conditions that existed together at a discrete moment in time. In terms of mid-Pliocene climate modelling studies using AGCMs, this does not present a significant problem. The PRISM reconstruction allows AGCMs to examine what a global average warm climate during the mid-Pliocene might have looked like [11–13]. However, outputs from AOGCMs have highlighted a clear disconnection between the proxy data, which are representative of a time slab, and relatively short model integrations that predict a climate state based on constant external forcing [17]. The motivation for defining a new time slice is the hypothesis that a component of this model–data inconsistency is related to the time slab nature of the proxy data.

While there have been a number of attempts to evaluate AOGCMs against the PRISM dataset, the fact that data and models are not reproducing the same objective, i.e. a discrete moment in time during the mPWP, makes the identification of any true model bias impossible [14,16,17,30]. In reality, a climate model simulation run for 1000 integrated years, using only a single realization of orbit, CO_2_ and other forcings, cannot reproduce a reconstruction of average warm climate conditions that is a product of multiple and changing/interacting forcing mechanisms.

What does this imply for previous mid-Pliocene-based estimates of Earth system sensitivity? Changes in the Earth's orbit are not relevant to calculations of either climate or Earth system sensitivity. If reconstructed changes in global ice volume or vegetation distribution are largely or even partly a function of orbital variability rather than CO_2_, then the utility of the mPWP for understanding the sensitivity of climate in the context of future climate change is diminished. Transient mid-Pliocene climate simulations using an Earth system model of intermediate complexity are becoming available. Here, CO_2_ forcing and orbital forcing have been imposed in isolation and in concert, and have suggested that a significant percentage of the additional feedback to global temperature derived from changes in vegetation cover and ice sheet extent are attributable to orbital forcing [39].

In summary, the PRISM time slab has given the scientific community insights into the nature of climate and environments of the time. However, the demands of modern data–model comparison indicate that progress in the future relies on the identification of a discrete time slice, or slices, for investigation within the Pliocene epoch.

## 2. Defining a new time slice(s)

### (a) Rationale and criteria for selection: where in the Pliocene?

**Figure 2.**
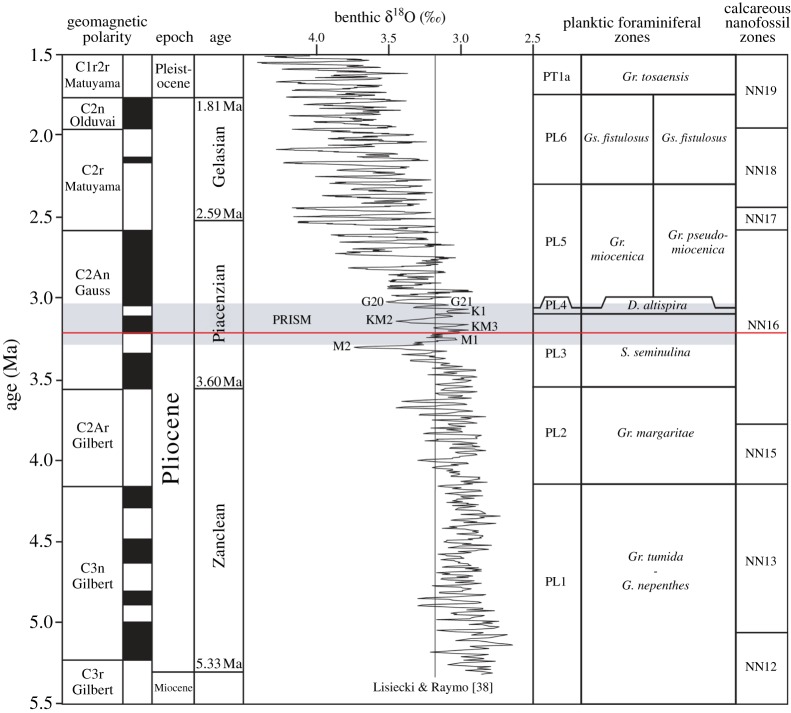
Position of the first Pliocene time slice (red line) and the PRISM time slab (grey-shaded band), relative to the geomagnetic polarity, magnetic reversals (black and white boxes), oxygen isotope stratigraphy (LR04 stack), planktic foraminiferal zones and calcareous nanofossil zones.

The benthic oxygen isotope record of Lisiecki & Raymo [38] (hereafter LR04) provides a view of changes in ice volume and bottom water temperature over the past 5 million years (figure 2). From the Pliocene section of the record, what interval of time should be selected to provide the focus for a new Pliocene time slice reconstruction? Ultimately, the selection depends on the scientific questions posed as well as the data required to effectively answer them.

Pragmatism suggests that the time slice is selected from within the existing PRISM time slab [1], as this provides the optimal starting point in terms of the availability of proxy data to underpin a new reconstruction. Choosing a time slice within the late, rather than early, Pliocene has added advantages in terms of reducing the potential for significant deviations in topography and ocean gateway configurations from present day. These factors cannot be easily determined (i.e. the Central America Seaway and the western cordillera of North and South America [40–42]), and therefore introduce unnecessary uncertainty into a climate model's experimental design. Identifying a time slice in the late Pliocene also reduces the potential for non-stationarity of environmental tolerance to bias geological proxies. In other words, the further back in time, the greater the potential for organisms/biota to have existed in different environments than they do today [43,44].

The PRISM project's aim is to understand environments and climates of a warmer world [45]. This scientific need has not diminished over the past 20 years; in fact, in the context of current estimates of future climate change, it is growing ever more acute [46]. Thus, a warm episode, defined by a negative benthic oxygen isotope excursion in the LR04 stack most likely representing a sea-level high stand, within the current PRISM time slab, is most appropriate for the selection of the first Pliocene time slice.

### (b) Rationale and criteria for selection: where in the PRISM time slab?

Given that the scenario of a discrete time slice falling on a biostratigraphic boundary or magnetic reversal is unlikely, identification will rely upon orbitally tuned high-resolution benthic oxygen isotope records. Assuming an equal availability of proxy data for any warm interval of the current PRISM time slab, the selection of which warm episode can be determined by a number of additional criteria. These criteria recognize the challenges of stratigraphically resolving a time slice, while at the same time attempting to reduce the uncertainty in both reconstructing and modelling the time slice. These include:

— selection of a negative oxygen isotope excursion of significant magnitude to identify an interval that was substantially warmer and had higher sea level than present day, and where the climate anomaly is significant producing a favourable signal-to-uncertainty ratio;
— selection of a time slice that falls at or very close to the peak in the identified benthic oxygen isotope excursion, to facilitate the time slice's identification in high-resolution benthic oxygen isotope records;
— selection of a negative oxygen isotope excursion of significant duration (thousands of years) to provide as large a time window as possible, facilitating correlation, and allowing the climate to respond sufficiently to the forcing in this interval; and
— selection of a time slice that is at or close to CO_2_ estimates from proxy records, to better constrain the range of CO_2_ values that should be imposed within climate models.

A careful examination of orbital parameters is warranted not just by the demands of chronology and correlation but also in terms of the forcing imposed within climate models. An immediate question emerges: what kind of orbital forcing should be imposed? For example, is a situation akin to the mid-Holocene or the Last Interglaciation required? In these cases, the response of climate models to a significant change in insolation at the top of the atmosphere (TOA) is studied [47]. Would a better result come from trying to identify a time slice that was warm and yet orbital forcing was the same, or very similar, to present day? If a warm episode within the current PRISM time slab can be identified, and it displays a modern or close to modern orbit, it removes or reduces an additional variable from the interpretation of the geological data and climate modelling results. It also simplifies the process of attributing what proportion of the global annual mean surface temperature increase, simulated by climate models, comes from different forcing mechanisms [48]. Finally, it enhances the potential for the time slice to provide more relevant information in the context of climate and Earth system sensitivity in the future, because the orbital forcing is the same as or very similar to present day. If an interval exists in which eccentricity, obliquity and precession do not vary substantially around a time slice, then orbital forcing will have a limited effect in creating variability in mean annual and seasonal temperatures. Focusing on such a time window would have the added advantage of helping to limit the impact on proxy temperature estimates of orbital variability, brought about by imperfect correlation to a time slice.

## 3. Astronomical solutions and orbital forcing

### (a) Astronomical solutions

To identify a warm episode within the existing PRISM time slab with modern or near modern patterns of insolation, it is necessary to calculate the planetary and precessional elements of the Earth for the entire time slab. Numerous astronomical solutions currently exist and provide the fundamental astronomical parameters of eccentricity, climatic precession and obliquity required for climate models [49]. The level of agreement that exists between solutions in calculating astronomical parameters for past periods in the Earth's history suggests that, as tools, they are sufficiently reliable to be used in palaeoclimate studies spanning the past 30 million years [49–51].

### (a) Orbital forcing through the PRISM time slab

#### (i) The La93 versus La04 orbital solution

**Figure 3.**
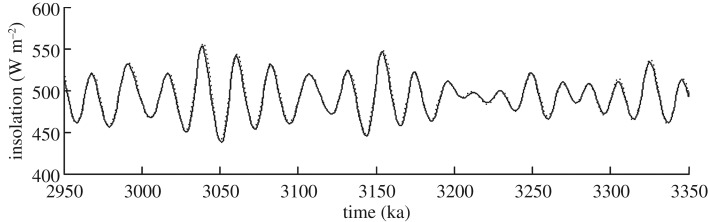
Comparison of insolation at 65°N on 21 June between the La93 (dotted curve) versus La04 (solid curve) orbital solutions between 2.95 and 3.35 Ma BP.

The LR04 stack [38] was developed using a nonlinear ice model that used insolation forcing derived from the Laskar *et al.* [51] (hereafter La93) astronomical solution. Since then, an updated version of the Laskar solution has been produced [49] (hereafter La04). The La04 solution has been improved with respect to La93 by using a direct integration of the gravitational equations for the orbital motion, and by improving the dissipative contributions, in particular, in the evolution of the Earth–Moon system [49]. Before the La04 solution can be used in concert with the LR04 stack to help identify a time slice(s) for reconstruction, we must determine that the solutions provided by La93 or La04 are the same or very similar. Figure 3 shows the difference between the two solutions at 65°N on 21 June (the forcing function used in the simple nonlinear ice model of LR04). During the PRISM time slab, the phasing between the two solutions is in strong agreement, as well as the magnitude of the insolation variation. Thus, we are confident in our use of the La04 solution to investigate orbital forcing during the PRISM time slab.

#### (ii) La04 reconstructions of insolation

**Figure 4.**
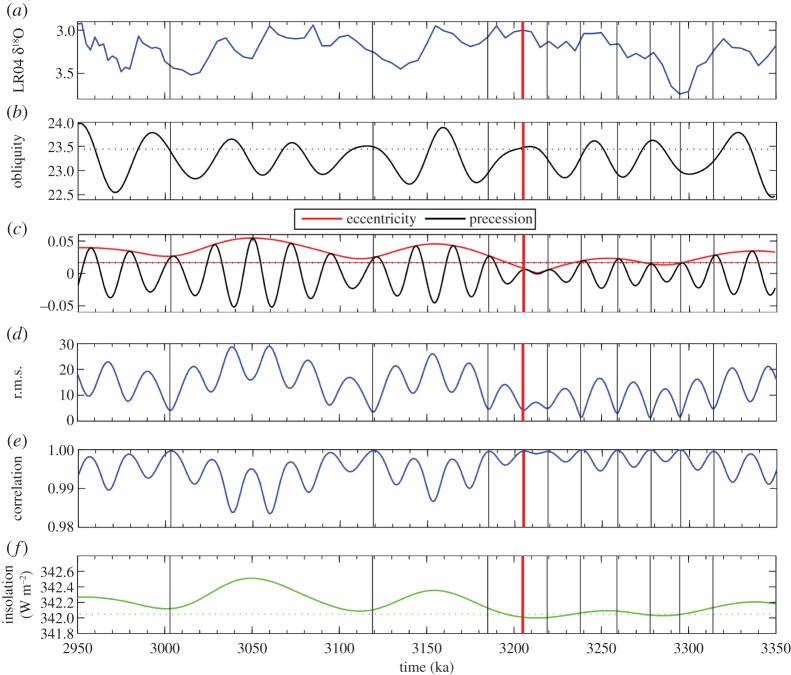
(*a*) The Lisiecki & Raymo [38] benthic oxygen isotope stack; (*b*) obliquity, with dashed horizontal line showing the present-day value; (*c*) precession and eccentricity as derived from the astronomical solution of Laskar *et al.* [49] (La04), with horizontal dotted black and solid red lines showing present-day values for eccentricity and precession; (*d*) the calculated r.m.s.e. (W m^−2^) and (*e*) correlation coefficient (0–1) for orbital solution considered for the Pliocene time slice; and (*f*) the variation in global mean TOA insolation according to La04, with the dotted horizontal green line denoting the modern value of global mean insolation. The vertical solid lines through each panel represent the best-fitting solutions considered in the study (black) and the discrete minimum in r.m.s.e. identified as the Pliocene time slice (solid red).

Variations in eccentricity, precession and obliquity according to La04 are shown in figure 4*b*,*c* for the period 2.95–3.35 Ma BP. This more than encompasses the PRISM time slab. A notable feature is a low in eccentricity values between 3.20 and 3.30 Ma, with correspondingly low modulations in precession. Across the PRISM time slab, insolation as a global annual mean derived from La04 varies by a maximum of 0.51 W m^−2^ (figure 4*f*). Largest variations are apparent younger than 3.2 Ma, with values that are generally closest to modern occurring prior to 3.2 Ma. We have also calculated the difference from present-day insolation at the TOA at each 1000 year time step between 2.95 and 3.35 Ma. This allows us to take into consideration how incoming insolation varies as a function of latitude and month in comparison with present day.

#### (iii) Statistical evaluation of La04 results

Our objective is to identify times within the PRISM time slab where the TOA insolation distribution is most similar to that of present day. In order to differentiate between the 400 insolation patterns produced, we evaluate the spatial similarity between the past and the present. The match between the spatial patterns has been evaluated in terms of correlation (*r*), root mean square (r.m.s.) difference and the ratio of the variances (standard deviation, s.d.). A perfect solution under this definition would have no error as computed by the r.m.s., would perfectly correlate with the present (*r*=1), and would have the same standard deviation.

We consider only solutions within the first 10 discrete minima in root mean square error (r.m.s.e.) as potential candidates for the first Pliocene time slice. This equates to an r.m.s.e. of less than 5 W m^−2^. R.m.s. offers the clearest distinction between the 400 potential solutions, as s.d. does not vary significantly among the ensemble. Each of the 10 defined minima in r.m.s.e. can include a number of individual orbital solutions that have very similar skill in matching the modern insolation distribution and are closely associated in time (table 1). Best-fitting orbital solutions from each discrete minima in r.m.s.e. are highlighted as vertical lines in figure 4.

**Table 1.** The age range of the 10 discrete minima in r.m.s.e. between 2.95 and 3.35 Ma, the best-fitting (closest match to modern insolation distribution at the top of the atmosphere, TOA) solution within each of the r.m.s. minima, the difference in global insolation (ΔINS) at the TOA at each best-fitting solution compared with modern, the root mean square error (r.m.s.), the correlation coefficient (CC) and the standard deviation (s.d.) from modern for each time point and an assessment of how well each discrete r.m.s. minimum matches the established criteria for the selection of the Pliocene time slice (see §2*a*). The discrete r.m.s. minimum highlighted in *italics* is encompassed by the selected interval time slice reconstruction.

r.m.s. minima (r.m.s. < 5 W m^−2^)	age range (ka)	best-fitting time point (ka)	ΔINS (W m^−2^)	r.m.s. (W m^−2^)	CC (0–1)	s.d. (W m^−2^)	description
1	3002–3004	3003	0.0532	4.1162	0.9997	158.4268	not situated at or near a discrete negative peak in LR04 stack
2	3118–3121	3119	0.0433	3.4920	0.9998	185.4160	not situated at or near a discrete negative peak in LR04 stack, but just above the base of the Kaena reversal (3116 ka;) in the Gauss Normality Chron (C2An.2n) [3]
3	3185–3186	3185	0.0606	4.6702	0.9996	158.3028	situated on a descending (towards positive) limb between two negative peaks
4	*3204–3207*	*3205*	−*0*.*0218*	*4*.*2657*	*0*.*9996*	*158*.*1467*	*centred on a broad peak (negative excursion), with Mammoth reversal (C2An.2r) directly before (3207 ka*) [3]
5	3219	3219	−0.0204	4.9019	0.9995	158.0499	within an isotopically light period, but on the falling limb with values becoming less negative
6	3236–3240	3238	0.0118	1.4689	1.0000	158.2245	in the transition zone towards a negative peak
7	3258–3260	3259	0.0296	2.9629	0.9998	158.1762	not situated at or near a discrete negative peak in LR04 stack
8	3276–3280	3278	−0.0070	1.1293	1.0000	158.2666	not situated at or near a discrete negative peak in LR04 stack
9	3293–3296	3295	−0.0014	1.4502	1.000	158.0723	situated at peak in positive isotopic excursion (M2 event)
10	3114	3314	0.0641	4.7294	0.9996	158.3965	outside of PRISM time slab and on a trend towards more positive isotope values

**Figure 5.**
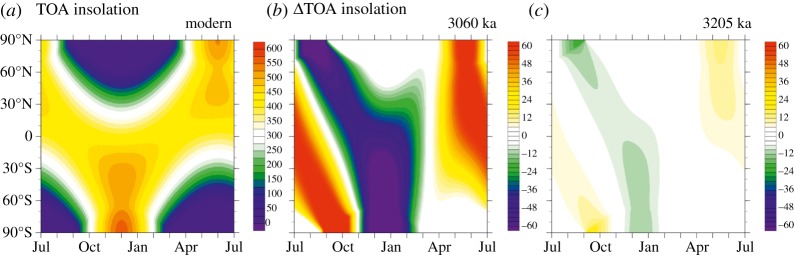
(*a*) Insolation distribution at the top of the atmosphere (TOA) in W m^−2^ for the modern; and the insolation anomaly between modern and (*b*) 3060 ka and (*c*) 3205 ka (derived from the La04 astronomical solution). The time 3060 ka is a time point during the PRISM time slab that exhibits the largest negative excursion in the benthic oxygen isotope record [38] (figure 4). The time 3205 ka is the time point identified in this study that satisfies the outlined criteria for being chosen as the Pliocene time slice.

Section 2*a* outlined the attributes that the chosen time slice should exhibit. Table 1 summarizes the relative attributes of the identified 10 discrete minima in r.m.s.e., as well as the best-fitting solutions. None of the best-fitting time solutions identified in our analysis is located at the lightest *δ*^18^O excursion seen in the LR04 stack for the PRISM time slab (figure 4*a*), as this is associated with a large change in orbital forcing from present day (figure 5*b*). Although there are multiple candidates for a Pliocene time slice reconstruction (e.g. within r.m.s.e. minima 5, 7 and 8; table 1), orbital solutions in the fourth discrete minimum in r.m.s.e. (3.204–3.207 Ma BP) provide the best overall solution given the rationale and criteria stated in §2.

### (c) Characteristics of the first Pliocene time slice

The chosen time slice sits in the normal polarity of the Gauss Chron between the Kaena (above) and Mammoth (below) reversals (figure 2). The peak deviation in benthic *δ*^18^O is centred on marine isotope stage (MIS) KM5c (or KM5.3). The 0.21–0.23‰deviation in *δ*^18^O could reflect a 21–23 m sea-level rise above modern (assuming 0.1‰equates to approx. 10 m of sea-level rise), providing that the signal is purely a function of ice volume rather than any change in deep-ocean temperatures. Assuming the near-total loss of the West Antarctic and Greenland ice sheets (a reasonable initial premise given proxy data and model outputs [18,20–22]), volume reduction from the East Antarctic ice sheet is a moderate 6 or 7 m of ice volume equivalent. This general interpretation of sea level from the LR04 stack is supported by a recent synthesis of sea-level records between 2.9 and 3.3 Ma BP by Miller *et al.* [23]. At approximately 3.205 Ma BP, the Miller *et al.* [23] synthesis indicates a maximum sea-level rise of 25±10 m (derived from Mg/Ca ratios of deep marine ostracods [52]). A mean of multiple sea-level records for approximately the same time indicate a peak sea-level rise of approximately 22±10 m.

During the time slice, incoming insolation is close to the modern distribution both seasonally and regionally (figure 4*c* and table 2). Eccentricity and precession are near zero, and obliquity remains near modern before and after the time slice. Therefore, the time slice is centred on an interval with a relatively stable orbit during which the distribution of insolation was close to modern (i.e. r.m.s.e. is low, and the correlation coefficient is high).

**Table 2.** The orbital parameters of eccentricity, precession and obliquity for modern and the Pliocene time slice (3.205 Ma BP) according to the astronomical solution of Laskar *et al.* [49].

time point	eccentricity	precession	obliquity (deg)
modern	0.016702	0.016280	23.4393
3205 ka	0.007483	0.006048	23.4736

Available proxy data for atmospheric CO_2_ (see reference [53] for a summary) places an upper limit of approximately 400 ppmv, with a cluster of four measurements within 100 ka of the time slice using three different proxy techniques (alkenones, boron isotopes and stomatal density) indicating a range between 300 and 380 ppmv. These concentrations are broadly supported by new high-resolution alkenone-proxy CO_2_ measurements presented by Badger *et al*. [54].

## 4. Current state of knowledge and future outlook

### (a) Availability of marine and terrestrial proxy data

**Figure 6.**
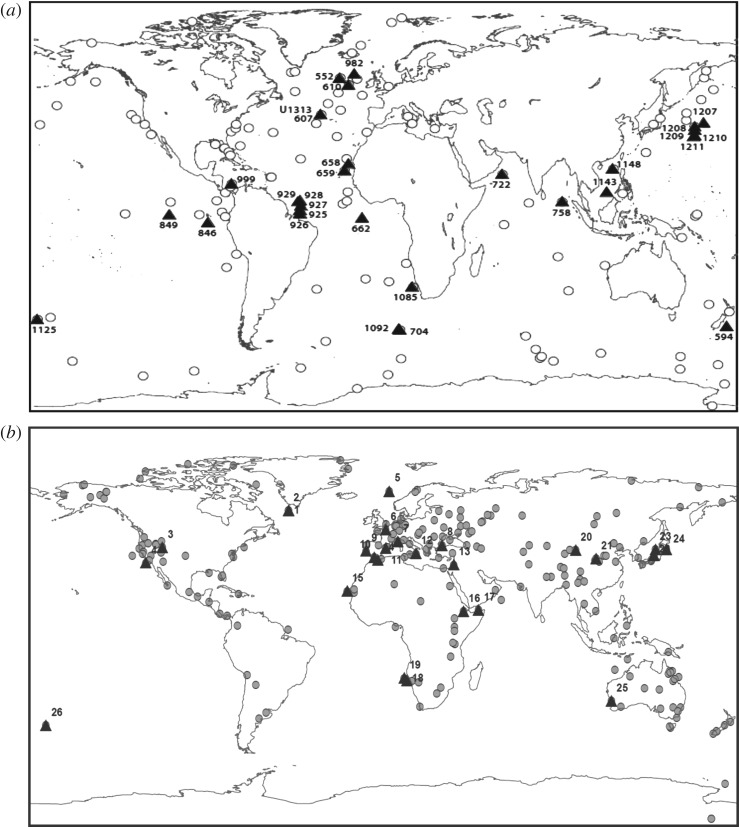
(*a*) Distribution of PRISM marine sites (open circles) and locations of potential time slice SST data (triangles). The existing PRISM time slab reconstruction (PRISM3D) is confined to a time slab with duration 240 ka, whereas the SST dataset currently in development (PRISM4) represents a significant development towards a time slice centred on MIS KM5c (KM5.3). (*b*) Distribution of PRISM3D terrestrial palaeobotanical sites (filled circles) and locations of *potential* time slice vegetation data (triangles).

**Figure 7.**
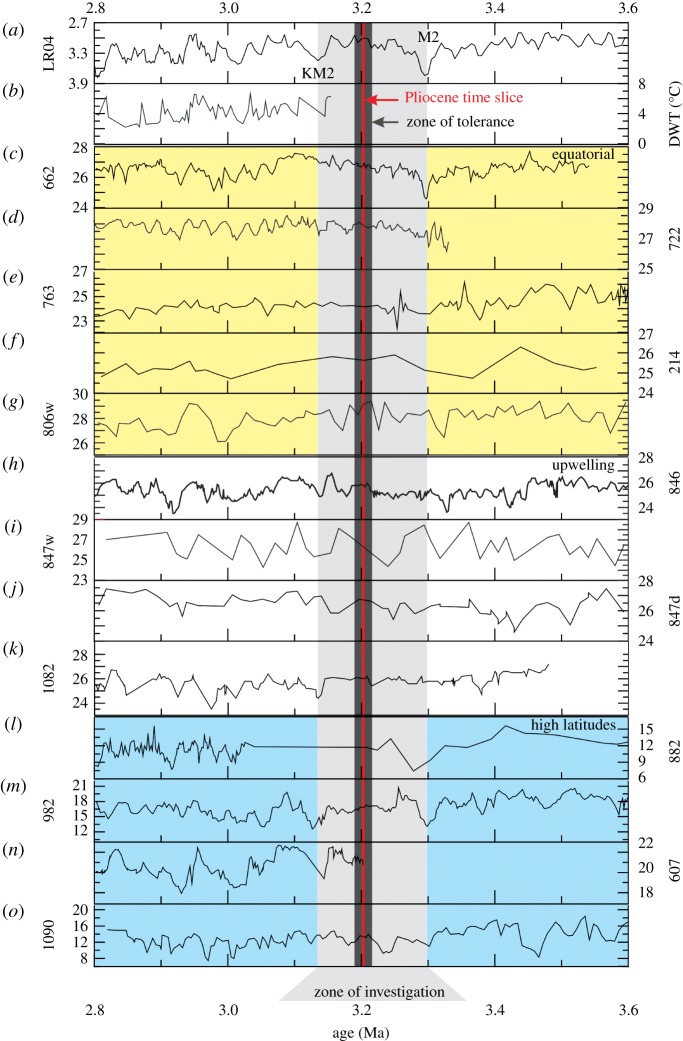
A compilation of published records of SST that span the late Pliocene and encompass the time slice study proposed here. All SST data are from IODP sites. The red line corresponds to the ideal target identified by the orbital forcing comparison (figure 5). The dark grey shading highlights a broader time window within which SST estimates could be derived and in all probability still reflect conditions during the time slice itself (*zone of tolerance*). The light grey shading highlights an interval for study to help identify the time slice in marine records, and also to understand climate variability before and after the time slice (*zone of investigation*). SST records (°C) are compared with (*a*) the benthic *δ*^18^O stack, LR04 [38] and (*b*) the deep-water temperature reconstruction from the North Atlantic site 607 [57]. Sites: (*c*) 662, Atlantic [58]; (*d*) 722, Arabian Sea [58]; (*e*) 763, Indian Ocean [59]; (*f*) 214, Indian Ocean [60]; (*g*) 806, West Pacific [61]; (*h*) 846, East Pacific [58]; (*i*) 847, East Pacific [61]; (*j*) 847, East Pacific [24]; (*k*) 1082, Southeast Atlantic [62]; (*l*) 882, Northwest Pacific 882 [63]; (*m*) 982, North Atlantic [64]; (*n*) 607, North Atlantic [65]; (*o*) 1090, Southern Ocean [63].

Recent advances in deep-sea drilling techniques have made possible the generation of numerous high-resolution orbitally tuned chronologies for Neogene marine sequences. Demand for finer-resolution deep-time palaeoclimate analysis makes this the norm rather than a rarity. The current PRISM SST dataset has 115 sites [17] (figure 6*a*) focused on a time slab of approximately 240 ka based on the warm peak averaging technique [35,55]. The next PRISM SST reconstruction, which is in development (PRISM4), represents more than a two order-of-magnitude increase in resolution with palaeoceanographic reconstruction 56. Preliminary analysis of available material for reanalysis from the PRISM project suggests that no fewer than 30 globally distributed SST sites may contribute to the first phase of time slice reconstruction for 3.204–3.207 Ma BP (figure 6*a* and table 3). These sites range from approximately 50° south to approximately 60° north latitude and sample all major ocean basins, with approximately half the sites confined to the low latitudes. In addition to the re-sampling of PRISM material, state-of-the-art high-resolution SST records, albeit of variable resolution, are available for the time slice in the published literature (figure 7). In total, 13 SST records are currently available sampling the high latitudes (IODP sites 1090, 607, 982 and 882), upwelling regions (IODP sites 1082, 847, 847 and 846) and equatorial regions (IODP sites 662, 722, 763, 214 and 806).

**Table 3.** Preliminary list of sites included in the existing PRISM time slab SST dataset capable of providing SSTs to support the new time slice reconstruction (figure 6*a*).

core site	latitude (°N)	longitude (°E)
DSDP 552	56.04	−23.23
DSDP 594	−45.52	174.95
DSDP 607	41.00	−32.96
DSDP 610	53.22	−18.89
ODP 658	20.75	−18.58
ODP 659	18.08	−21.03
ODP 662	−1.39	−11.74
ODP 704	−46.88	7.42
ODP 722	16.62	59.80
ODP 758	5.38	90.37
ODP 846	−3.09	−90.82
ODP 849	0.18	−110.52
ODP 925	4.20	−43.49
ODP 926	3.72	−42.91
ODP 927	5.47	−44.48
ODP 928	5.46	−43.75
ODP 929	5.98	−43.74
ODP 982	57.52	−15.87
ODP 999	12.74	−78.74
ODP 1085	−29.37	13.99
ODP 1092	−46.41	7.08
ODP 1125	−42.55	−178.17
ODP 1143	9.36	113.29
ODP 1148	18.84	116.57
ODP 1207	37.79	162.75
ODP 1208	36.13	158.20
ODP 1209	32.65	158.51
ODP 1210	32.22	158.26
ODP 1211	32.00	157.85
IODP U1313	41.00	−32.96

Salzmann *et al.* [8] describe terrestrial proxy data, 202 globally distributed sites, which were synthesized to create a global land-cover reconstruction for the entire Piacenzian Stage. Figure 6*b* and table 4 show the distribution of 26 terrestrial localities from the original dataset of 202 sites, which potentially may be able to provide vegetation data to evaluate climate model predictions for the Pliocene time slice. In reality, correlating terrestrial data to any Pliocene time slice is not possible with the same degree of confidence as the marine proxy data. This will require consideration when terrestrial data–model mismatches are highlighted.

**Table 4.** Preliminary list of terrestrial sites included in the PRISM time slab dataset [8] *potentially* capable of providing vegetation data to support the new time slice reconstruction (figure 6*b*).

map ID	site	latitude (°N)	longitude (°E)
1	ODP 646, Labrador Sea	58.22	−48.20
2	ODP 646, Leg 105	58.21	−48.37
3	Great Salt Lake, UT	41.00	−112.50
4	DSDP 467, Leg 63	33.85	−120.76
5	ODP 642, Norwegian Sea	67.22	2.94
6	La Londe, Normandy	49.31	0.95
7	Alpes-Maritimes	43.82	7.19
8	DSDP 380, LEG 42B	42.10	29.61
9	Rio Maior	39.35	−8.93
10	Andalucia G1	36.38	−4.75
11	Tarragona	40.83	1.13
12	Bianco/Bovalino	38.25	16.40
13	Hula Basin	33.00	35.60
14	Nador	35.18	−2.93
15	ODP 658, Cape Blanc	20.75	−18.58
16	Hadar	11.29	40.63
17	DSDP 231, Leg 24	11.89	48.25
18	DSDP 532, Leg 75	−21.09	14.46
19	ODP 1082	−21.10	11.82
20	Yumen, Jiuxi Basin	39.78	97.53
21	Xifeng, Loess Plateau	35.88	107.97
22	Himi Area, Toyama	37.15	137.25
23	ODP 794A	40.19	138.22
24	DSDP 440B/438A	40.00	143.60
25	Yallalie, Perth	−30.43	115.77
26	ODP 1123, Leg 181	−41.78	−171.50

In the marine realm, a plausible strategy for identifying the time slice would be initially to identify the MIS M2 and sample forward in time (i.e. produce a time series) at the highest practical sampling resolution in each core. MIS KM2 provides another isotopic marker useful for reference after the time slice itself. We term this interval between MIS M2 and KM2 the *zone of investigation* (figure 7). Limitations in correlation may create situations in which multiple temperature estimates can be plausibly attributed to the time slice. In such circumstances, the appropriate information from the point of view of data–model comparison is the range in absolute reconstructed temperatures (or range in temperature differences) rather than an average. If a multi-proxy approach is adopted, then the range in temperature estimates from each proxy method should be clearly stated.

### (b) Enduring uncertainties: challenges and new opportunities

While the identification of discrete time slices reduces variability in proxy climate data used to evaluate models, and will place tighter constraints on the design of climate model experiments, it is not a panacea for the Pliocene. Moving to a time slice will lead to a reduction in the amount and geographical spread of proxy data available for data–model comparison, particularly in the terrestrial realm. Issues of bioturbation, varying accumulation rates and the potential for different proxy methods to monitor different parts of the water column in different parts of the year all remain [55]. Furthermore, while the selection of the first Pliocene time slice was partly based on the fact that the interval will minimize the potential bias introduced by orbital forcing, it does not remove it entirely (figure 5*c*). This means that orbital forcing will change to a degree through and around the studied time slice. Therefore, time slice sensitivity experiments with climate models are warranted to fully explore orbital influences on regional climates.

**Figure 8.**
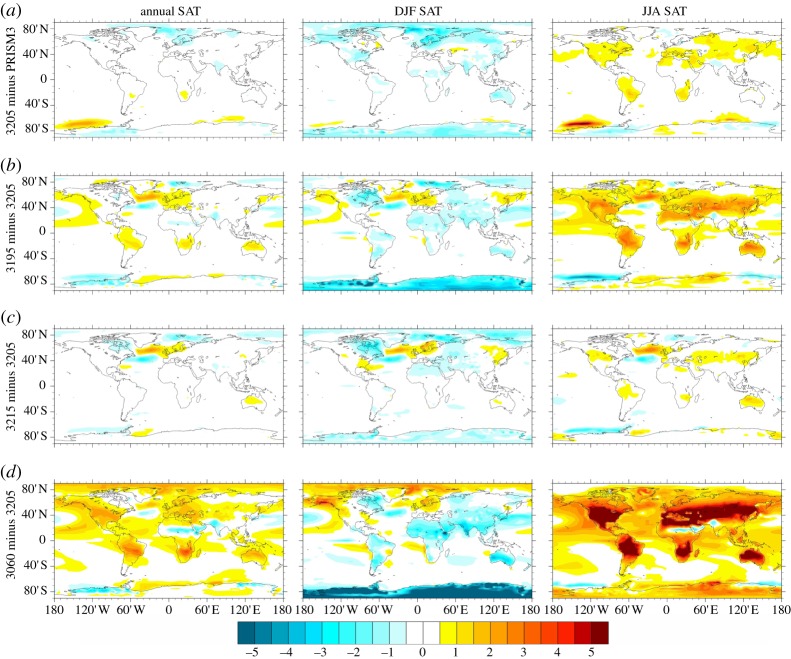
Annual mean and seasonal mean (December, January and February, DJF; June, July and August, JJA) Pliocene surface air temperature predictions from HadCM3. (*a*) Identified time slice minus a Pliocene experiment with a modern orbital configuration (PRISM3). Pliocene experiments given orbital configurations appropriate to (*b*) 3195 and (*c*) 3215 ka BP (3195 and 3215 ka minus 3205 ka BP). (*d*) An experiment for the MIS K1 PlioMAX super interglacial event minus the identified time slice at 3205 ka BP characterized by a near modern orbital configuration.

To provide an initial assessment of the degree to which differences in insolation calculated for the 3205 ka BP time slice compared with modern can affect a climate model's simulation of Pliocene climate, we show the difference in mean annual as well as seasonal average surface air temperatures (SATs) between two Pliocene simulations using the Hadley Centre coupled climate model version 3 (HadCM3; figure 8). The deviation in SATs as an annual and seasonal mean is no more than 1°C in most ocean and terrestrial regions. The majority of the differences are not statistically significant at a 95% confidence interval (CI).

To provide an initial assessment of how stable climate could have been in response to orbital forcing around the time slice itself, we show results from two further sensitivity studies in which the model has been run with orbital forcing equivalent to 3195 and 3215 ka BP, 10 ka either side of the identified time slice at 3205 ka BP. Compared with mean annual SATs simulated for the time slice, simulations for 3195 and 3215 ka BP rarely differ by more than 1°C. The predicted differences are normally insignificant at a 95% CI. One exception to this is in the North Atlantic, where differences reach 2–3°C and are statistically significant (figure 8). The pattern of SAT anomalies is akin to a North Atlantic Oscillation (NAO) dipole, and the results even appear to show the Pacific branch of the Arctic Oscillation (AO). This suggests a few scenarios for the genesis of the changes in the North Atlantic. They may represent a temporal shift of normal NAO during model spin-up that is not removed by the *t*-test because of long-period oscillations. Alternatively, they may represent changes in modes of interannual variability, or be indicative of significant orbital impact on NAO. Providing that these differences are not a model or statistical artefact, the results imply that in the North Atlantic correlation to the time slice would have to be better than 10 ka to keep orbital forcing biases on temperature to less than 3°C. Seasonally larger changes that are statistically significant are predicted: for example, 3°C over Antarctica during the Southern Hemisphere summer and up to 3°C over land in the simulation for 3195 ka in the Northern Hemisphere summer (figure 8). These seasonal differences will not affect proxy temperature estimates if the proxy itself truly provides an estimate of mean annual temperature. However, they should be considered in data–model comparisons if a proxy technique has the potential to be biased to a temperature reconstruction for a particular season. Therefore, the selection of the time slice and its characteristic stability in orbital forcing immediately before and after creates a time window in which palaeotemperature information can be imperfectly correlated to the time slice itself, but may still be more or less representative of the general conditions that existed during the time slice. We term this a *zone of tolerance* (figure 7).

To place these differences in climate due to orbital variability around the Pliocene time slice in context, we have performed a final experiment with HadCM3 in which an orbital forcing appropriate to 3060 ka was prescribed. The 3060 ka PlioMAX peak (or super interglacial event) is characterized by one of the lightest benthic oxygen isotope excursions evident in the entire PRISM time slab (MIS K1) [66]. The time 3060 ka BP is characterized by the La04 orbital solution as displaying a dramatically different profile of insolation by month and latitude compared with either present day or the identified Pliocene time slice at 3205 ka BP (figure 5). It is also an interval in which the total amount of insolation as a global annual mean differs from present day, or from the 3205 ka BP time slice, by +0.5 W m^−2^ (figure 4). Figure 8 shows the model-predicted differences in annual and seasonal mean SAT for 3060 ka BP compared with the Pliocene time slice at 3205 ka BP. As an annual mean, SAT differences can exceed +3°C and are almost always statistically significant at a 95% CI. This general increase in mean annual temperature is partly caused by the 0.5 W m^−2^ enhancement in annual global mean insolation calculated for 3060 ka BP compared with 3205 ka BP. It is also strongly influenced by much larger changes in seasonal insolation patterns and SATs, which often exceed +5°C, particularly during the Northern Hemisphere summer months (June–August) over the land. If any proxy data included in either the PRISM3D marine or terrestrial environmental reconstructions are actually representative of 3060 ka BP, then it would not be expected to concur with model simulations for the Pliocene set up with a modern, or essentially modern, profile of insolation. This analysis also suggests that 3060 ka BP is inappropriate as a means to assess climate or Earth system sensitivity due to the significant orbital overprint on SATs (figures 5 and 8).

Even with greater certainty in the orbital forcing given to models for the Pliocene, many of the challenges in deriving certain boundary conditions for models remain constant across a time slab or time slice approach. Perhaps the most challenging is the initial state of the ice sheets. The time slice approach also means that the application of a time slab-based vegetation reconstruction as a boundary condition becomes more difficult to justify, implying that future experiments for time slices during the Pliocene will be increasingly dominated by coupled ocean–atmosphere–vegetation models, where vegetation is a predicted rather than a prescribed element.

Ultimately, given the uncertainties in prescribed forcing, even for defined time slices, only a limited amount of information can be gained by comparing only one realization of Pliocene climate from a climate model to proxy data. A comprehensive programme of well-justified time slice sensitivity experiments with climate models is required and can be examined in concert with the proxy data during future data–model comparisons. The number of sensitivity experiments that are likely to be required for a Pliocene time slice will be less than the requirements of the current PRISM time slab. Nevertheless, the number required will remain demanding computationally, even for full complexity climate models of intermediate resolution. Therefore, other techniques to sufficiently explore uncertainty space with climate models, such as a Latin hypercube approach that has been successfully applied in palaeoclimate research [67], may be required. The implementation of such a strategy will generate progressively more rigorous data–model comparisons, where an identified signal or residual may highlight a deficiency in climate model predictions for the Pliocene with greater confidence.

Finally, from the point of view of understanding the Pliocene, it is essential to develop a better appreciation of how climate varied through time. We have identified other time slices prior to 3.2 Ma BP that provide potential targets for environmental reconstruction. Of particular interest is the evolution of Pliocene climate and environments from the M2 to KM2 ‘glacial’ events (the *zone of investigation* identified in figure 7). Until more is understood about how climate evolved towards and away from the Pliocene time slice, we will not be able fully understand what the time slice represents. Through increasing our understanding of the nature and variability of Pliocene climates, we can understand the Pliocene world more completely, and, at the same time, apply the Pliocene as a test for models used to predict future climate change with increasing certainty.

## 5. Conclusions

In this study, we outlined the rationale and criteria for the definition of a discrete time slice for environmental reconstruction during the mPWP. The mPWP time slab concept, developed by the US Geological Survey PRISM project, has provided a means to explore and understand climate and environments of a warm phase in Earth history in considerable detail. However, a change in methodology to time slice reconstructions, which have been used so successfully in the Quaternary, is necessary to reduce uncertainties in environmental reconstruction as well as climate/environment modelling. While a range of time slices should be studied that examine different facets of Pliocene climate (e.g. periods with strong orbital forcing or Pliocene ‘glacial’ events), the highest initial priority is to examine a warm period in which orbital forcing was the same as or very similar to present day. This is justifiable given the current requirements to better understand climate and Earth system sensitivity, and to robustly evaluate models used for climate change prediction.

A suitable time slice representative of a warm event or ‘interglacial’ within the existing PRISM time slab has been identified through the calculation and statistical evaluation of orbital forcing using the La04 orbital solution. The time slice is centred on a negative peak (0.21–0.23‰) in the LR04 benthic oxygen isotope stack at MIS KM5c (KM5.3) at 3.204–3.207 Ma BP. Limits of chronology and correlation mean that the time slice may not be resolved in marine records from different ocean basins to a window of only a few thousand years. However, between 3.215 and 3.195 Ma BP, orbital forcing was similar to present day. Atmospheric CO_2_ may have peaked at approximately 400 ppmv, with CO_2_ proxies supporting a common range of between 300 and 380 ppmv. While challenges and uncertainties will remain from a modelling and environmental reconstruction standpoint, the reduced temporal range of a time slice facilitates the construction of more focused sensitivity studies using climate models. Time slices are also short enough to contemplate performing fully transient simulations with a full-complexity intermediate-resolution climate model in the future.
